# Health-related quality of life and subjective well-being among children aged 9–12 years in Shandong Province, China

**DOI:** 10.1186/s12955-024-02258-7

**Published:** 2024-05-31

**Authors:** Zhao Shi, Aihua Cao, Shunping Li, Jianglin Wang, Jin Zhang, Julie Ratcliffe, Gang Chen

**Affiliations:** 1 Centre for Health Management and Policy Research, School of Public Health, Cheeloo College of Medicine, Shandong University, Jinan, China; 2 NHC Key Lab of Health Economics and Policy Research, Shandong University, Jinan, China; 3 Center for Health Preference Research, Shandong University, Jinan, China; 4 Department of Pediatric, Qilu Hospital, Shandong University, Jinan, China; 5 Shandong Electric Power Central Hospital, Jinan, China; 6 Qingdao Municipal Hospital, Qingdao, China; 7 College of Nursing and Health Sciences, Flinders University, Adelaide, Australia; 8 Centre for Health Economics, Monash Business School, Monash University, Melbourne, Australia

**Keywords:** Children, Health-related quality of life, Subjective well-being, CHU9D, PedsQL™, SLSS

## Abstract

**Purpose:**

To investigate the health-related quality of life (HRQoL) and subjective well-being (SWB) of children aged 9–12 years in eastern China, and examine concordance within child self-reported and parent proxy-assessed.

**Methods:**

Data was collected from 9 to 12 years old children (including their parents) in Shandong Province in 2018. Participants self-completed a hard-copy questionnaire including Child Health Utility 9D (CHU9D), Pediatric Quality of Life Inventory (PedsQL)™ 4.0 Short Form 15 Generic Core Scales (hereafter the PedsQL™), Student’s Life Satisfaction Scale (SLSS), as well as information on socio-demographic characteristics and self-report health status. Spearman’s correlation coefficients and the difference between sub-groups were conducted to assess and compare the agreement on HRQoL and SWB instruments. Exploratory factor analysis (EFA) was used to ascertain the number of unique underlying latent factors that were associated with the items covered by the two generic HRQoL and the SWB instruments. The concordance of child self-reported and parent proxy-assessed was analyzed using weighted kappa coefficient and Bland-Altman plots.

**Results:**

A total of 810 children and 810 parents were invited to participate in the survey. A valid sample of 799 (98.6%) children and 643 (79.4%) parents completed the questionnaire. The child self-reported mean scores were CHU9D = 0.87, PedsQL™ = 83.47, and SLSS = 30.90, respectively. The parent proxy-assessed mean scores were PedsQL™ = 68.61 and SLSS = 31.23, respectively. The child self-reported PedsQL™ was moderately correlated with the CHU9D (*r* = 0.52). There was a weak correlation between CHU9D and SLSS (*r* = 0.27). The EFA result found 3 factors whilst seven SLSS items grouped into a standalone factor (factor 3), and the nine dimensions of CHU9D shared two common factors with the PedsQL™ (factor 1 and factor 2). A low level of concordance was observed across all comparisons and in all domains (weighted kappa < 0.20) between parents and their children. Furthermore, a high level of discordance was observed between child self-reported and father proxy-assessed.

**Conclusions:**

CHU9D and PedsQL™ instruments have a higher agreement in measuring the HRQoL in children. CHU9D/PedsQL™ and SLSS instruments showed a low agreement and EFA result suggested that measuring SWB in children potentially may provide further information, which might be overlooked by using HRQoL instruments exclusively. Concordance of child self-reported and parent proxy-assessed was poor. Overall, mother-child concordance was higher than father-child concordance.

**Supplementary Information:**

The online version contains supplementary material available at 10.1186/s12955-024-02258-7.

## Introduction

Health-related quality of life (HRQoL) is generally considered to be a multifactorial construct that focuses on individuals’ subjective evaluation of their physical health, mental health and social functioning [1]. The assessment of HRQoL forms a vital component for the economic evaluation of healthcare, medical regulatory and health insurance reimbursement decisions [2, 3]. Economic evaluations of children’s healthcare and public health interventions are receiving increasing interest internationally and in China [4, 5]. Adolescence is characterized by dynamic brain and physical development, exploration of sexual identity, and drive for independence and greater autonomy [6–8]. During the transitional phase from childhood to adulthood, children and adolescents encounter many challenges, mainly including unhealthy lifestyles, risky behaviors, as well as emerging mental health issues [8, 9]. Moreover, adolescence is regarded as a critical development stage for achieving human potential [8]. Hence, investments in children and adolescents’ health bring benefits today, into future adult life, and for the next generation [8, 10].

HRQoL instruments are classified into non-preference-based and preference-based measures (PBMs) [2]. Non-preference-based measures are widely used in paediatric populations and are premised on simple summary scoring of individual items or dimensions to generate HRQoL scores [11]. However, PBMs are required for economic evaluation [12, 13]. In cost-utility analysis (CUA), the commonly used benefit measure is quality-adjusted life years (QALYs), which accounts for both the length and quality of life. Quality of life is measured by using health state utility (HSU) scores, which lie on a (0–1) scale where 1 and 0 represent full health and being dead, respectively [2, 14]. In recent years, increasing attention has been focused on the development and application of children-specific preference-based HRQoL instruments [11, 12].

Assessment of health status in children differs from adults and requires a different conceptual approach due to rapid rates of development, dependency on parents/caregivers and differences in disease epidemiology [4, 15]. Among a total of nine available preference-based HRQoL instruments globally for application with paediatric populations, the Child Health Utility 9D (CHU9D) is unique in that it represents the only generic preference-based paediatric HRQoL instrument to date developed exclusively from its inception with young people [13, 16]. The non-preference-based HRQoL instruments frequently employed in paediatric health research and evaluations is the Pediatric Quality of Life Inventory (PedsQL)™ 4.0 Short Form 15 Generic Core Scales (hereafter the PedsQL™) [17]. It has both the child self-report and the parent proxy-report versions available and has demonstrated satisfactory psychometric properties [17].

Life satisfaction is a subjective evaluation of the overall quality of life and is considered to be the key indicator of subjective well-being (SWB) [18]. SWB can be defined as an individual’s cognitive evaluation of life, the presence of positive emotions and the lack of negative emotions are commonly [18, 19]; it goes beyond health and has gained increasing attention in policy debates in recent years [20, 21]. The Student’s Life Satisfaction Scale (SLSS) is a brief youth SWB instrument and has been translated into different language versions [22–24]. However, to date the focus in the literature on SWB has mainly been in adult populations rather than children [25].

Proxy assessment, whereby a close caregiver (e.g., a parent and/or healthcare professionals provide on-going care and support to the child) assess the child’s HRQoL and/or SWB on their behalf, which is a common approach adopted in child populations [26]. However, the concordance between proxy-reported and self-reported outcomes in children is mixed [27–29]. On the one hand, self-report could be problematic because the reliability of children’s judgments may be questioned [4]; on the other hand, proxy-report can also be biased, involving both under-estimation and over-estimation of children’s health [30].

Trends in health outcome measurement beyond the commonly used instruments of HRQoL, and encompassing a broader range of generic outcomes including both health and well-being in recent years [21]. Some studies have shown a higher or lower HRQoL level of concordance/discordance between children and their proxies both in the healthy population and in that with special health features [26, 28, 31–33]. However, the relationships between HRQoL and SWB and the level of concordance/discordance between children and their proxies in assessments of HRQoL and SWB have not been studied previously in China. In addition, previous exploratory factor analysis (EFA) studies have indicated that CHU9D [34] and SLSS [24, 35] only one factor emerged, and four factors were extracted from the PedsQL™ [36]. Further research is needed to explore whether potential for different underlying structure between HRQoL and SWB instruments in youth population. Therefore, the first aim of this study is to assess and compare the agreement of HRQoL and SWB based on a large school-based survey in children aged 9–12 years old in Shandong province, China. The second aim is to explore the concordance between parental proxy assessment and child assessments dyad reports on HRQoL and SWB.

## Methods

### Participants and procedures

A cross-sectional survey was conducted with primary school students aged 9–12 years old as well as their parents in a period prior to the commencement of the COVID-19 pandemic (June to August 2018) in Shandong Province, China. Shandong Province is located in the east of China, with a population of about 100 million [37]. In 2021, the gross regional product of Shandong Province amounted to RMB 7312.9 billion (US$ 1,135.7 billion), ranking the third-largest economy in China [37].

A stratified and cluster sampling method was applied to select the study population from different geographic areas of Shandong Province. Three tiers were applied to ensure the representation of our sample. Firstly, we selected three prefecture-level cities in the east (Yantai), middle (Jinan) and south (Linyi) of Shandong Province. Secondly, three counties were selected respectively based on their economic and social development level, and then three public primary schools from these three counties were selected. Finally, two classes (each with around 45 students) were randomly chosen from grades 4 to 6 in each school. There were 6 classes surveyed in each school, and a total of 810 students were expected to be surveyed. Respondents were included if they: (1) were primary school students aged 9–12 years old, (2) were the father or mother of the student, (3) gave informed consent, (4) were literate and had no disease that limited cognitive function. Respondents were excluded if they: (1) did not agree to participate in the investigation among child’s guardian, (2) were guardians other than parents.

Researchers underwent training sessions prior to the survey. All the students in the school on the day of the survey completed the questionnaire independently in the classroom, and parents were consulted during parent-teacher conferences. Firstly, the researchers explained the meaning of the survey and the requirements to fill in the questionnaire. Then participants completed the questionnaire by themselves. Researchers would give an explanation if they had any semantic or conceptual understanding issues when completing the questionnaires. The children were asked to complete a survey that including the CHU9D, PedsQL™, SLSS instruments and sociodemographic variables (gender, age, residence and whether the only child). Parents’ survey contains their own and their spouse’s education level as well as proxy assessment of their children’s PedsQL™ and SLSS. The CHU9D was not included in the parents, because at the time of study design the Chinese version of the proxy-assessed CHU9D questionnaire was not yet available.

The study was approved by the Qilu Hospital, Shandong University (Reference No. 2018178), and the research adhered to the tenets of the Declaration of Helsinki. The investigation was conducted with the consent of the head teacher at all participating schools in advance. Written informed consent was obtained from both participating children and their parents. Participants were informed about their freedom of refusal. If they feel uncomfortable, they can withdraw from the survey at any time.

### Instruments

We used Child Health Utility 9D (CHU9D) as a generic preference-based instrument to measure HRQoL of children. CHU9D is the only instrument that was developed from its inception exclusively for application with young people [38], it has adequate psychometric properties and is wildly used among children and adolescents [12]. The self-reported and proxy-assessed short form (15 items) version of PedsQL™ and Student’s Life Satisfaction Scale (SLSS) were used to evaluate the HRQoL and SWB of children, respectively. The short form (15 items) version of PedsQL™ characterized by its brevity, availability of age-appropriate versions and parallel forms for child and parent [17]. SLSS is a brief and psychometrically acceptable instrument of youth SWB [39], and the Chinese version has been validation [23].

### CHU9D

The Child Health Utility 9D (CHU9D) is a generic preference-based HRQoL instrument designed specifically for using in the economic evaluation of healthcare interventions in children and adolescents [16, 40]. The CHU9D instrument contains 9 dimensions: worried, sad, pain, tired, annoyed, schoolwork/homework, sleep, daily routine and ability to join in activities; each dimension contains 5 severity levels. The CHU9D has been validated for self-completion by young people (aged 7–17 years) [15, 16]. CHU9D is the original UK English questionnaire, as well as Chinese, Spanish, Welsh, Dutch, Italian, Japanese, Danish, French, Canadian, Swedish and Portuguese translations available [41]. The Chinese version of CHU9D is a valid and reliable instrument to measure HRQoL for children and adolescents in China [42]. In this study, the CHU9D was only used in children and the Chinese-specific scoring algorithm was used [43]. The Cronbach’s alpha (α) and McDonald’s omegas (ω) of the CHU9D in the current study were 0.75 and 0.75, respectively.

### PedsQL™

The Pediatric Quality of Life Inventory (PedsQL)™ 4.0 Short Form 15 Generic Core Scales (PedsQL™) is a brief, 15-item version of the 23-item PedsQL™ 4.0 Generic Core Scale [17, 44]. It encompasses four subscales: Physical Functioning (5 items), Emotional Functioning (4 items), Social Functioning (3 items), and School Functioning (3 items). To calculate PedsQL™ dimension and total scores, items were linearly transformed into a 0-100 scale with the higher scores indicating the better HRQoL. Then the dimension/total score was computed as the mean score of relevant item scores. The PedsQL™ instrument has appropriate reliability and validity in both patient and healthy populations [17]. The Mandarin Chinese (Traditional) version of the PedsQL™ 4.0 Short Form 15 Generic Core Scales is a relatively reliable and valid instrument [45]. This study conducted a survey in mainland China (Shandong province), so the Mandarin Chinese (Simplified) version of the self-reported and proxy-assessed PedsQL™ questionnaires were adopted in this study [46]. Cronbach’s alpha (α) and McDonald’s omegas (ω) for the PedsQL™ were all above 0.7 in both self- and proxy-assessed, except for the social functioning dimension (Supplementary Table 1).

### SLSS

The Student’s Life Satisfaction Scale (SLSS) is developed for children and adolescents from 8 to 18 years old to evaluate overall life satisfaction [22, 47, 48]. It consists of seven items: (1) My life is going well; (2) My life is just right; (3) I would like to change many things in my life; (4) I wish I had a different kind of life; (5) I have a good life; (6) I have what I want in life; (7) My life is better than most kids. Participants were asked to respond to each item on a six-point scale ranging from ‘strongly agree’ to ‘strongly disagree’. Item 3 and Item 4 are negatively worded and require reverse coding in the scoring. Possible scores for the SLSS range from 7 to 42, with a higher score indicating a greater level of life satisfaction [24]. The psychometric evaluation of the Chinese version of SLSS has been verified [23], and the 7-item Chinese version of the SLSS questionnaires were adopted in this study [23]. The Cronbach’s alpha (α) of the self- and proxy-assessed SLSS version in the current study were 0.68 and 0.66, and the McDonald’s omegas (ω) were 0.57 and 0.52, respectively.

### Data analysis

Descriptive analyses including means, standard deviation (SD), median, and the interquartile range (IQR) were reported. The floor or ceiling effects were considered to be present if more than 15% of the respondents achieved the lowest or highest possible score, respectively [49]. The normality test was used for the Shapiro-Wilk test. To compare different children socio-demographic characteristics scores, the non-parametric tests (i.e., Mann-Whitney U test and Kruskal Wallis test) and Cohen effect size (*d*) were used to examine sub-group differences for CHU9D, PedsQL™ and SLSS scores. The two-tailed *P* < 0.05 was considered statistically significant. According to the following cut offs: Cohen’s *d* < 0.2 = small; 0.2 < Cohen’s *d* < 0.5 = moderate; Cohen’s *d* ≥ 0.5 = strong, Cohen’s *d* ≥ 0.8 large [50]. Cronbach’s α coefficient and McDonald’s omegas (ω) were applied to estimate the internal consistency.

To assess and compare the agreement on HRQoL and SWB instruments, Spearman’s correlation coefficient was used to examine the correlations between instruments. Correlations less than 0.3 were considered weak, 0.3–0.7 moderate and > 0.7 strong [51]. Mean PedsQL™ and SLSS scores were also calculated and reported for each level of CHU9D response. To further explore the discrepancy/correlation in the descriptive systems between the two HRQoL and SWB instruments, EFA was conducted. EFA was used to ascertain the number of unique underlying latent factors that were associated with the items covered by the two generic HRQoL and the SWB instruments. Despite the conceptual origins of different instruments, EFA is a commonly adopted strategy to examine different instruments whether to share the same set of the underlying factors or measure separate constructs [52–54]. The Bartlett’s test of sphericity (*P* < 0.05) and a Kaiser Meyer-Olkin (KMO) measure of sampling adequacy reaching ≥ 0.50 would be considered appropriate to conduct EFA [55]. The factors were extracted by the maximum likelihood (ML) method. ML estimation provides foundations for hypothesis testing, including tests for the number of factors. Although it is commonly thought to be a disadvantage that ML estimation explicitly assumes that the sample is from a multivariate normal distribution, ML estimation of factor structure is fairly robust against departures from normality [56]. The number of factors to be extracted was determined using the parallel analysis [57]. Rotation was performed using the promax method to allow for potential correlations among the factors.

The concordance within parent-child was analyzed using weighted kappa coefficient with Landis and Koch’s criteria and Bland-Altman plots [56]. The levels of concordance were judged as: slight: < 0.20; fair: 0.21–0.40; moderate: 0.41–0.60; substantial: 0.61–0.80; and almost perfect: 0.81-1.00 [58].

With the exception of the Bland-Altman plot and EFA, which were conducted using MedCalc version 16.8 and FACTOR 12.03.02 software for Windows [59], respectively. Internal consistency of the instruments was conducted using SPSS version 27. All other statistical analyses were performed using Stata version 14.1.

## Results

### Respondent characteristics

A total of 810 children and 810 parents were invited to participate in the survey. Child self-reported data from eleven participates (1.4%) were subsequently excluded due to invalid or missing responses among HRQoL/SWB instruments, and providing a final study sample for the data analysis of 799 (98.6%) children. 167 parents had missing responses, a valid sample of 643 (79.4%) parents completed the proxy survey. Detailed socio-demographic characteristics of the participants are presented in Table 1. Among the 799 children, 50.4% were boys, 44.3% were only-child, and 61.6% of the children resided in cities. Among the 643 parents, 60.8% were mothers, and 22.2% (of mothers) /25.0% (of fathers) have completed undergraduate or higher education, respectively.

**Table 1:** Participants characteristics

Characteristics	Analysis sample
**Panel A: Children**	*N* = 799 (%)
Age	
9	72 (9.0)
10	244 (30.5)
11	305 (38.2)
12	178 (22.3)
Gender	
Boys	403 (50.4)
Girls	396 (49.6)
Resident	
Urban areas	492 (61.6)
Rural areas	307 (38.4)
Only-child	
Yes	354 (44.3)
No	445 (55.7)
Self-assessed health status	
Excellent	381 (47.7)
Very good	234 (29.3)
Good	128 (16.0)
Fair or poor	56 (7.0)
**Panel B: Parents**	*N* = 643 (%)
Parent	
Mother	391 (60.8)
Father	252 (39.2)
Mother’s education level *	
Primary school and below	83 (12.9)
Middle school	187 (29.1)
High school	147 (22.9)
Junior college	83 (12.9)
Undergraduate and above	143 (22.2)
Father’s education level *	
Primary school and below	31 (4.8)
Middle school	207 (32.2)
High school	156 (24.3)
Junior college	88 (13.7)
Undergraduate and above	161 (25.0)

* Parents reported their own and their spouse’s education level

### Agreement on HRQoL and SWB instruments

**Fig. 1:**
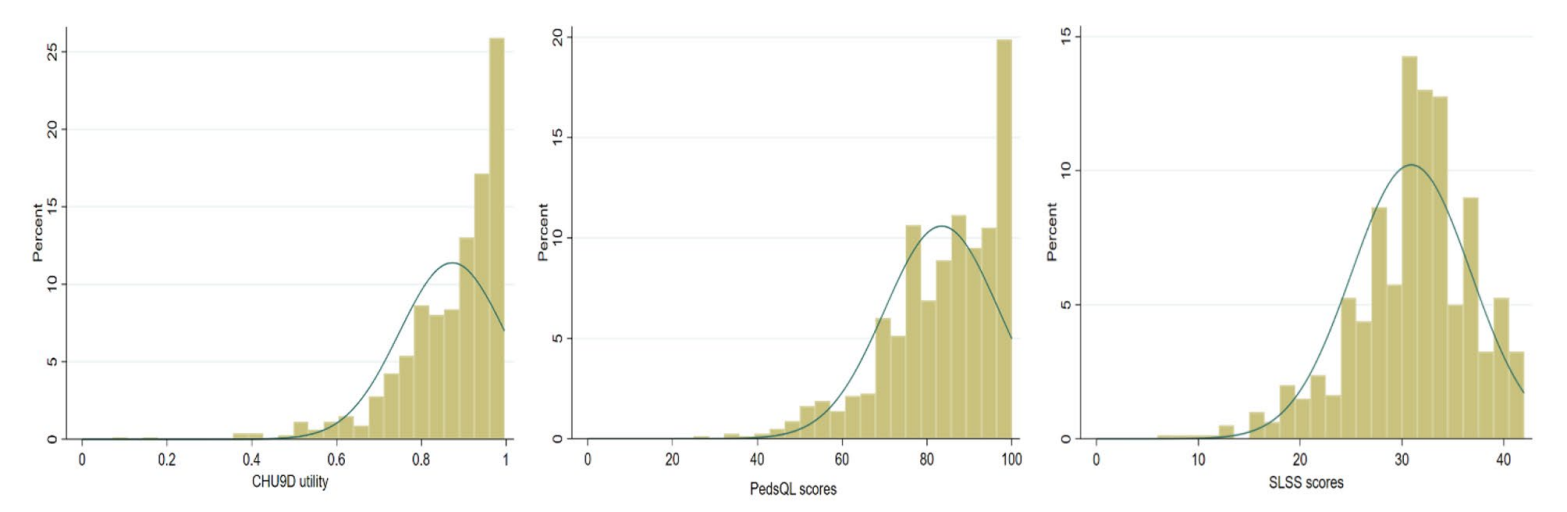
Distribution of health-related quality of life and subjective well-being scores

In this school-based sample, 93% reported themselves as having excellent, very good or good health (Table 1). The mean (SD) of the CHU9D utility was 0.87 (0.12). The mean (SD) of the PedsQL™ and SLSS scores were 83.47 (13.45) and 30.90 (5.85) for children, respectively (Table 2). Figure 1 shows the distribution of CHU9D, PedsQL™ and SLSS, respectively.

**Table 2:** Comparison children self-reported scores of the CHU9D, PedsQL™ and SLSS

Measures	Theoretical range	Observed range	Mean (SD)	Ceiling effect *N* (%)	Floor effect *N* (%)
CHU9D	0.06, 1	0.10, 1	0.87 (0.12)	171 (21.4)	0 (0)
PedsQL™	0, 100	25, 100	83.47 (13.45)	85 (10.6)	0 (0)
SLSS	7, 42	7, 42	30.90 (5.85)	18 (2.3)	1 (0.1)

PedsQL™: Pediatric Quality of Life Inventory (PedsQL)™ 4.0 Short Form 15 Generic Core Scales; SLSS: Student’s Life Satisfaction Scale; CHU9D: Child Health Utility 9 Dimension instrument. Ceiling effect, 15% of respondents scored the highest possible health/subject well-being state; Floor effect, 15% of respondents scored the lowest possible health/subject well-being state

Table 3 shows Spearman’s correlation coefficients (*r*) among child self-reported HRQoL and SWB outcome measures; among them, the strength of correlation between two HRQoL instruments was the strongest (*r* = 0.52). Between HRQoL and SWB, it was moderately correlated between PedsQL™ and SLSS (*r* = 0.30), whilst a low correlation was found between CHU9D and SLSS (*r* = 0.27).

**Table 3:** Spearman’s correlations between children self-reported CHU9D and SLSS (*N* = 799)

Dimensions/Total scores	CHU9D										SLSS
	Worried	Sad	Pain	Tired	Annoyed	School work /homework	Sleep	Daily routine	Activities	CHU9D Utility	
PedsQL™											
Physical Functioning	0.13	0.12	0.21	0.18	0.17	0.22	0.22	0.19	0.13	0.35	0.24
Emotional Functioning	0.29	0.23	0.29	0.30	0.29	0.27	0.29	0.15	0.09	0.50	0.34
Social Functioning	0.23	0.15	0.25	0.21	0.20	0.28	0.19	0.16	0.06	0.38	0.31
School Functioning	0.21	0.14	0.20	0.16	0.18	0.34	0.22	0.15	0.11	0.37	0.27
Total scores	0.28	0.21	0.30	0.29	0.27	0.34	0.29	0.20	0.13	0.52	0.30
SLSS	0.11	0.07	0.12	0.06	0.13	0.11	0.18	0.15	0.09	0.27	-

PedsQL™: Pediatric Quality of Life Inventory (PedsQL)™ 4.0 Short Form 15 Generic Core Scales; SLSS: Student’s Life Satisfaction Scale; CHU9D: Child Health Utility 9 Dimension instrument

The KMO was 0.881 for pooled CHU9D, PedsQL™, and SLSS items, Bartlett’s test of sphericity coefficient was 8255.7 (*P* ≤ 0.001), suggesting that the data were appropriate to conduct EFA. EFA further explores the discrepancy/correlation in the descriptive systems between two HRQoL and SWB instruments (Supplementary Table 2). The EFA result found 3 factors whilst seven SLSS items grouped into a standalone factor (factor 3), and the nine dimensions of CHU9D shared two common factors with the PedsQL™ (factor 1 and factor 2). As shown in Supplementary Table 3, three factors were extracted based on the parallel analysis, and their absolute value of correlations ranged from 0.434 (between factors 2 and 3) to 0.593 (between factors 1 and 2).

Table 4 summarizes the differences of the children self-reported CHU9D, PedsQL™ and SLSS scores between sub-groups based on children characteristics. Statistically significant differences were found for all three instruments by self-assessed health status (that poor self-reported health status was significantly associated with lower HRQoL and SWB scores). Furthermore, significant differences were observed in gender (girls had higher scores than boys based on CHU9D), resident status (children from urban China had higher scores than from rural China according to CHU9D and PedsQL™), and whether respondents were the only child of the family (i.e., been the only child of the family had higher scores based on PedsQL™).

**Table 4:** Differences of the children self-reported CHU9D, PedsQL™ and SLSS scores between sub-groups based on children characteristics (*N* = 799)

Characteristics	CHU9D			PedsQL™			SLSS	
	Mean (SD)	Median (IQR)		Mean (SD)	Median (IQR)		Mean (SD)	Median (IQR)
Gender								
Boys	0.86 (0.14)	0.90 (0.79–0.96)		82.44 (14.24)	85.00 (73.33–93.33)		30.54 (6.00)	32 (27–35)
Girls	0.88 (0.11)	0.91 (0.82–0.96)		84.53 (12.54)	86.67 (76.67-95.00)		31.28 (5.69)	32 (28–35)
*P*-value ^a^	**0.04**			0.07			0.190	
Effect size (*d*)	-0.18			-0.16			-0.13	
Resident								
Urban areas	0.88 (0.13)	0.91 (0.82–0.99)		86.26 (12.22)	88.33 (80.00–95.00)		31.13 (6.09)	32 (28–35)
Rural areas	0.86 (0.12)	0.89 (0.80–0.95)		79.02 (14.15)	81.67 (70.00–90.00)		30.56 (5.46)	31 (27–34)
*P*-value ^a^	**< 0.001**			**< 0.001**			0.067	
Effect size (*d*)	0.13			0.56			0.10	
Only-child								
Yes	0.88 (0.13)	0.91 (0.81–0.99)		85.53 (12.25)	88.33 (78.33-95.00)		31.27 (6.07)	32 (28–35)
No	0.87 (0.12)	0.90 (0.81–0.95)		81.48 (14.15)	85.00 (73.33–93.33)		30.62 (5.67)	32 (27–34)
*P*-value ^a^	0.31			**< 0.001**			0.057	
Effect size (*d*)	0.03			0.28			0.11	
Self-assessed health status								
Excellent	0.91 (0.11)	0.94 (0.86–0.99)		87.96 (11.28)	90.00 (81.67–96.67)		31.76 (5.52)	32 (29–35)
Very good	0.87 (0.11)	0.89 (0.80–0.95)		82.42 (12.77)	84.17 (75.00-91.67)		30.85 (5.39)	31 (28–34)
Good	0.83 (0.13)	0.84 (0.76–0.93)		77.34 (13.39)	76.67 (70.00-88.33)		29.38 (6.13)	31 (26–34)
Fair or poor	0.78 (0.19)	0.84 (0.70–0.92)		71.24 (16.31)	73.33 (60.00-83.33)		28.86 (7.93)	30 (24–34)
*P*-value ^b^	**< 0.001**			**< 0.001**			**< 0.001**	

PedsQL™: Pediatric Quality of Life Inventory (PedsQL)™ 4.0 Short Form 15 Generic Core Scales; SLSS: Student’s Life Satisfaction Scale; CHU9D: Child Health Utility 9 Dimension instrument. ^a^ Mann–Whitney U test; ^b^ Kruskal–Wallis test. Bold values indicate the *P* < 0.05

### Concordance of within parents-child

As shown in Table 5, the mean PedsQL™ score reported by parents (68.61 ± 18.63) was significantly lower than the mean scores self-reported by children (83.47 ± 13.45). On the contrary, the proxy-assessed mean SLSS score was slightly higher than the self-reported scores of children (31.23 ± 4.57 vs. 30.90 ± 5.85). At the dimension level, the lowest score reported by parents was physical function (64.12 ± 28.83), while the lowest score reported by children was emotional function (74.80 ± 20.50). Mothers reported the lowest scores in the physical functioning dimension (65.33 ± 28.57) and the highest scores in the social functioning dimension (75.72 ± 19.54), while a similar pattern is found in father-reported results. It can be further found that the scores reported by mothers were much closer to the self-reported scores by children. In general, these results showed that there were statistically significant differences in PedsQL™ and SLSS scores between self-reported and parent-reported (*P* < 0.01).

**Table 5:** PedsQL™ and SLSS scores by respondents (Mean, SD)

Dimensions/Total scores	Children (*n* = 799)	Mothers (*n* = 391)	Fathers (*n* = 252)	Parents (*n* = 643)
PedsQL™				
Physical Functioning	86.72 (15.75)	65.33 (28.57)	62.26 (29.19)	64.12 (28.83)
Emotional Functioning	74.80 (20.50)	71.46 (18.97)	72.69 (20.35)	71.94 (19.52)
Social Functioning	88.98 (15.62)	75.72 (19.54)	73.47 (19.91)	74.84 (19.70)
School Functioning	84.12 (18.92)	66.85 (22.28)	63.22 (22.56)	65.43 (22.44)
Total scores	83.47 (13.45)	69.35 (18.30)	67.48 (18.10)	68.61 (18.63)
SLSS	30.90 (5.85)	31.59 (4.52)	30.66 (4.59)	31.23 (4.57)

PedsQL™: Pediatric Quality of Life Inventory (PedsQL)™ 4.0 Short Form 15 Generic Core Scales; SLSS: Student’s Life Satisfaction Scale. SD: standard deviation

The level of concordance between parents and children with PedsQL™ and SLSS is presented in Table 6. The weighted kappa coefficient was used to further test concordance on dyads. All the *P*-values were statistically significant, except for the SLSS scores between fathers and children. A low level of concordance was observed across all PedsQL™ total scores (weighted kappa < 0.20). Mother-child concordance was slightly higher than that of fathers on most dimensions except for physical function. The concordance between parents and children was the highest in the school functional dimension (weighted kappa = 0.080), and the lowest in the emotional functional dimension (weighted kappa = 0.044). The patterns were the same for either parent.

**Table 6:** Level of agreements between parents- and children-reported scores

Dimensions/Total scores	Father-children (*n* = 178)			Mother-children (*n* = 302)			Parent-children (*n* = 480)		
	Weighted kappa	*P*		Weighted kappa	*P*		Weighted kappa		*P*
PedsQL™									
Physical Functioning	0.063	< 0.001		0.052	0.008		0.058		< 0.001
Emotional Functioning	0.040	0.017		0.051	0.022		0.044		0.002
Social Functioning	0.059	0.006		0.081	0.002		0.067		< 0.001
School Functioning	0.072	< 0.001		0.094	< 0.001		0.080		< 0.001
Total scores	0.023	0.009		0.032	0.003		0.026		< 0.001
SLSS	0.024	0.062		0.021	0.001		0.039		< 0.001

PedsQL™: Pediatric Quality of Life Inventory (PedsQL)™ 4.0 Short Form 15 Generic Core Scales; SLSS: The Student’s Life Satisfaction Scale

**Fig. 2:**
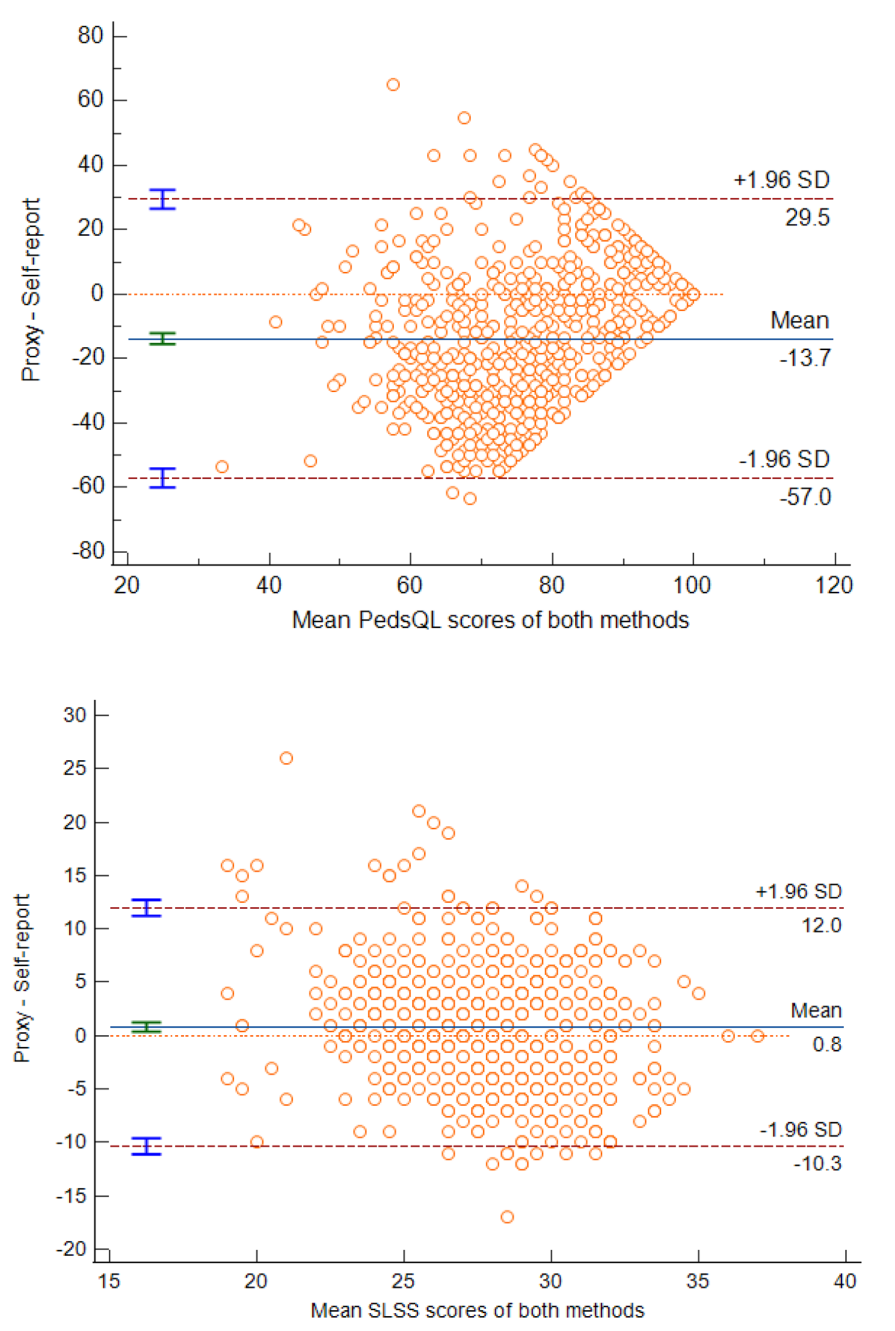
Bland-Altman plots of comparison among children and parents. The 95% limits of agreement are shown with a dashed line and the mean difference between both measurements is shown with a solid line

Bland-Altman plots (Fig. 2) further showed that for each of the two instruments among parents and children measures the range of 95% limits of agreement (LOA) were 86.5 (children & parents with PedsQL™) and 22.3 (children & parents with SLSS), respectively.

## Discussion

Focusing on a school sample in eastern China, this study compared the differences in measuring HRQoL and SWB of children aged 9–12 years using three different outcome instruments. To the best of our knowledge, this is the first empirical study to provide HRQoL and SWB concordance data between children and their parents based on a large school-based survey in China.

The study sample is similar to a previous school-based survey using the same instrument in China, a mean CHU9D utility of 0.87 was found in our study compared with a mean of 0.85 from a sample of 1,912 students (aged 8–17 years old) in Baoji City, western China [42]. A recent study conducted on the relationship between the lifestyle-related behaviors and HRQoL of Chinese children (aged 9–17) reported a lower mean CHU9D utility of 0.78, and the result showed that lifestyle-related behaviors may have an additive effect on young population HRQoL [60]. For PedsQL™ instruments, this study found the mean PedsQL™ scores for the boys and girls were 82.44 and 84.53, which was consistent with the previous study of 8–12 years old boys (84.30) and girls (85.74) in Taiwan, China [45]. The total SWB scores (30.90) in this study are similar to a previous study (29.32) that described the development of the Portuguese version of the SLSS and the examination of its psychometric properties [24].

Overall, the findings indicated that both the child self-reported PedsQL™ was moderately correlated with the CHU9D (*r* = 0.52), and the results confirmed the relatively high agreement between the two instruments. The findings of the two instruments in this Chinese student sample were similar to the main findings from a previous Australian study (*r* = 0.63) [61]. At the dimension level, where different concepts were measured, a moderate degree of correlation between similar dimensions was found between the two instruments. The advantage of using a preference-based measure (CHU9D) when assessing HRQoL in this adolescent age group is that it facilitates the calculation of QALYs for health economic analyses in the form of CUA, which plays an important role in health system decision making [40].

Our results found a low agreement between HRQoL (especially the HSU) and SWB instruments. The empirical evidence from the EFA further suggested that the two instruments are complementary rather than substitutable. Based on item loadings, this study found that seven SLSS items grouped into a standalone factor (SWB), the CHU9D shared two common factors with the PedsQL™ (HRQoL). Compared with these instruments’ previous validation studies [24, 34–36], the EFA findings provide new information for different underlying structure. The EFA unanimously showed that the dimensions measured by SWB always fell into a separate factor. This suggests that measuring SWB in children and adolescents potentially may provide further information that might not be captured by the application of HRQoL instruments exclusively, which is consistent with studies in adults [52, 62].

With the developments in childhood theory and children’s rights legislation [63], children’s SWB is currently routinely measured and incorporated into decision-making processes in the health system [20, 25, 64]. Previous study showed that SWB overlaps with HRQoL and picks up the broader impacts of healthcare [20, 65]. There is a burgeoning interest in the measurement of SWB for informing health and social care decision-making [21, 66]. It was found that the factors influence adult SWB and children’s SWB are distinct, and such as age micro-level factors influence the children’s SWB [67, 68]. Although a few SWB instruments (SWLS, BMSLSS, etc.) have been validation in children and adolescents [69], research in this area to date has been hampered by a lack of reliable and valid instruments of positive SWB appropriate for children, especially in cross-cultural studies [67, 70].

A low level of concordance has also been described between parents and children in this study conducted using the HRQoL and SWB instruments. In terms of proxy- versus self-reports, one review conducted by Jiang et al. proved a child’s perception of HRQoL differed from their parents, with parents frequently underestimating HRQoL for severe diseases such as meningitis, asthma, lung disease, while proxies tended to report higher HRQoL than children themselves for mild health conditions such as the general population, overweight or obesity [28]. As observed with the PedsQL™ instrument in study, which showed low concordance in the emotional between parents and children. Although the level of concordance between mothers and children was low in our study, it was found that mother-child dyads showed a higher concordance than fathers-children’s dyads, and those results are similar to this study [71]. In Chinese culture setting, mothers tend to take the family as the center and take more responsibility for children’s education and other work [72, 73], so they may have a better understanding of children’s health.

From the findings of this study, we suggest that the HRQoL and SWB should be assessed by children and obtain more comprehensive results. Previous research also recommends driving the inclusion of children in self-reporting their own HRQoL wherever possible and limiting the reliance on proxy reporting of children’s HRQoL [33]. In reality, some children may be unable to self-assess their own HRQoL due to limited cognitive capability and the reading level in reality. Proxy reports (e.g., parent/guardian or a health professional) are usually used to assess the child’s HRQoL when self-reports are not feasible. In order to avoid proxy reports from the proxy’s personal judgment, clear instructions incorporating the child’s perspective into the assessment could be developed for all proxy types, which would improve the quality and accuracy of proxy-assessed HRQoL and/or SWB assessments. Furthermore, mothers’ proxy assessment maybe a priority alternative in the Chinese culture context.

There are several limitations to this study. Firstly, the patients were recruited from eastern China and the vast majority of participants in this study are Han Chinese, so the conclusions may not be applicable to the whole Chinese population. Secondly, this was a cross-sectional study and it will be important to further assess the changes in HRQoL using a longitudinal survey design. Thirdly, parents did not complete the CHU9D instrument in this study, which would limit the comparison of HSU between parents and children. In addition, owing to missing values, there were fewer matched parent-child observations than the sample size of the children sample. Last but not least, the use of the Cohen effect size (*d*) and ML method in this case despite the deviation from normal distribution, they have been wildly used in some PRO research [74, 75]. Future studies could use different methods to validate the findings of this paper.

## Conclusion

The study found that children aged 9–12 in eastern China had a good overall HRQoL and SWB. HRQoL and SWB instruments showed a low agreement and complementary relationship, which suggests that measuring SWB in children potentially may provide further information that might not be captured through the application of HRQoL instruments exclusively. Besides, a low level of concordance has been described between parents and children in this study conducted using the HRQoL and SWB instruments. Although the level of concordance between mothers and children was low in our study, these dyads showed a higher concordance than fathers-children’s dyads.

### Electronic supplementary material

Below is the link to the electronic supplementary material.

Supplementary Material

## Data Availability

The datasets used and analyzed during this study are available from the corresponding author on reasonable request.

## Abbreviations

CHU9D: Child Health Utility 9D
CUA: Cost-utility analysis
EFA: Exploratory factor analysis
HRQoL: Health-related quality of life
HSU: Health state utility
IQR: Interquartile range
LOA: Limits of agreement
ML: Maximum likelihood
PBMs: Preference-based measures
PedsQL™: Pediatric Quality of Life Inventory™ Version 4.0 Short Form Generic Core Scales
QALYs: Quality-adjusted life years
SD: Standard deviation
SLSS: Student’s Life Satisfaction Scale
SWB: Subjective well-being
