# Supplementary material for: Health-related quality of life and subjective well-being among children aged 9–12 years in Shandong Province, China
Source: Health Qual Life Outcomes. 2024 May 31;22:41. doi: 10.1186/s12955-024-02258-7 (PMC11140898; doi:10.1186/s12955-024-02258-7)
Supplement: Supplementary file 1 — Supplementary Material [file 12955_2024_2258_MOESM1_ESM.docx]

**Health-related quality of life and subjective well-being among children aged 9–12 years in** **Shandong province, China**

Zhao Shi ^1,2,3^, Aihua Cao ^4^, Shunping Li ^1,2,3^, Jianglin Wang ^5^, Jin Zhang ^6^, Julie Ratcliffe ^7^, Gang Chen ^8^

1. Centre for Health Management and Policy Research, School of Public Health, Cheeloo College of Medicine, Shandong University, Jinan, China
2. NHC Key Lab of Health Economics and Policy Research (Shandong University), Jinan, China
3. Center for Health Preference Research, Shandong University, Jinan, China
4. Department of Pediatric, Qilu Hospital, Shandong University, Jinan, China
5. Shandong Electric Power Central Hospital, Jinan, China
6. Qingdao Municipal Hospital, Qingdao, China
7. College of Nursing and Health Sciences, Flinders University, Adelaide, Australia
8. Centre for Health Economics, Monash Business School, Monash University, Melbourne, Australia

**Corresponding author** Shunping Li, E-mail: [lishunping@sdu.edu.cn](mailto:lishunping@sdu.edu.cn).

Zhao Shi and Aihua Cao have contributed equally to this work.

**Journal name：**Health and Quality of Life Outcomes

**Supplementary Table**

**Supplementary Table 1 Internal consistency of the Chinese mandarin version of the PedsQL™**

| Dimensions/Total scores | Children self-reported version | | Parents proxy-assessed version | |
| --- | --- | --- | --- | --- |
|  | Cronbach’s alpha (α) | McDonald’s omegas (ω) | Cronbach’s alpha (α) | McDonald’s omegas (ω) |
| Physical Functioning | 0.74 | 0.74 | 0.91 | 0.92 |
| Emotional Functioning | 0.81 | 0.81 | 0.83 | 0.83 |
| Social Functioning | 0.69 | 0.69 | 0.49 | 0.50 |
| School Functioning | 0.79 | 0.79 | 0.78 | 0.86 |
| Total score | 0.86 | 0.86 | 0.90 | 0.90 |

PedsQL™: Pediatric Quality of Life Inventory (PedsQL)™ 4.0 Short Form 15 Generic Core Scales

**Supplementary Table 2 Exploratory factor analysis comparing the CHU9D, PedsQL™ and SLSS**

| Measures | Items/dimensions | Factor | | |
| --- | --- | --- | --- | --- |
|  |  | 1 | 2 | 3 |
| CHU9D | Tired | 0.632 |  |  |
| CHU9D | Annoyed | 0.627 |  |  |
| CHU9D | Sad | 0.624 |  |  |
| CHU9D | Worried | 0.601 |  |  |
| PedsQL™ | Sad or blue | 0.553 |  |  |
| PedsQL™ | Angry | 0.507 |  |  |
| PedsQL™ | Worry about what will happen to me | 0.482 |  |  |
| CHU9D | Daily routine | 0.441 |  |  |
| CHU9D | Sleep | 0.426 |  |  |
| PedsQL™ | Afraid or scared | 0.421 |  |  |
| CHU9D | Pain | 0.391 |  |  |
| PedsQL™ | Trouble keeping up with my school-work |  | 0.840 |  |
| PedsQL™ | Hard to pay attention in class |  | 0.800 |  |
| PedsQL™ | Forget things |  | 0.691 |  |
| PedsQL™ | Do chores around the house |  | 0.562 |  |
| PedsQL™ | Walk 100 meters |  | 0.554 |  |
| PedsQL™ | Do sports activity or exercise |  | 0.457 |  |
| PedsQL™ | Have trouble getting along with other teens |  | 0.447 |  |
| PedsQL™ | Run |  | 0.435 |  |
| PedsQL™ | Lift something heavy |  | 0.411 |  |
| PedsQL™ | Other teens do not want to be my friend |  | 0.402 |  |
| CHU9D | Schoolwork/homework |  | 0.310 |  |
| PedsQL | Other teens tease me |  | 0.305 |  |
| CHU9D | Ability to join in activities |  | [0.233] |  |
| SLSS | My life is going well |  |  | 0.873 |
| SLSS | My life is just right |  |  | 0.870 |
| SLSS | I have a good life |  |  | 0.810 |
| SLSS | I have what I want in life |  |  | 0.516 |
| SLSS | My life is better than most kids |  |  | 0.339 |
| SLSS | I would like to change many things in my life ^a^ |  |  | [0.111] |
| SLSS | I wish I had a different kind of life ^a^ |  |  | [0.112] |

Extraction Method: Maximum Likelihood. Rotation Method: Promax. Loadings smaller than 0.3 are not shown in the table. PedsQL™: Pediatric Quality of Life Inventory (PedsQL)™ 4.0 Short Form 15 Generic Core Scales; SLSS: Student’s Life Satisfaction Scale; CHU9D: Child Health Utility 9 Dimension instrument.

^a^: Items are reverse-scored.

**Supplementary Table 3 Factor Correlation Matrix**

| Factor | 1 | 2 | 3 |
| --- | --- | --- | --- |
| 1 | 1.000 |  |  |
| 2 | 0.593 | 1.000 |  |
| 3 | -0.508 | -0.434 | 1.000 |

Extraction Method: Maximum Likelihood.

Rotation Method: Promax.
